# Association between gingivitis and anterior gingival enlargement in subjects undergoing fixed orthodontic treatment

**DOI:** 10.1590/2176-9451.19.3.059-066.oar

**Published:** 2014

**Authors:** Fabricio Batistin Zanatta, Thiago Machado Ardenghi, Raquel Pippi Antoniazzi, Tatiana Militz Perrone Pinto, Cassiano Kuchenbecker Rösing

**Affiliations:** 1 Associate professor, Department of Stomatology, Federal University of Santa Maria (UFSM).; 2 Assistant professor, Franciscano University Center (UNIFRA).; 3 Postdoc in Periodontics, Adjunct professor, Federal University of Rio Grande do Sul, (UFRGS).

**Keywords:** Epidemiology, Orthodontics, Gingivitis, Gingival enlargement

## Abstract

**Objective:**

The aim of this study was to investigate the association among gingival enlargement (GE), periodontal conditions and socio-demographic characteristics in subjects undergoing fixed orthodontic treatment.

**Methods:**

A sample of 330 patients undergoing fixed orthodontic treatment for at least 6 months were examined by a single calibrated examiner for plaque and gingival indexes, probing pocket depth, clinical attachment loss and gingival enlargement. Socio-economic background, orthodontic treatment duration and use of dental floss were assessed by oral interviews. Associations were assessed by means of unadjusted and adjusted Poisson's regression models.

**Results:**

The presence of gingival bleeding (RR 1.01; 95% CI 1.00-1.01) and excess resin around brackets (RR 1.02; 95% CI 1.02-1.03) were associated with an increase in GE. No associations were found between socio-demographic characteristics and GE.

**Conclusion:**

Proximal anterior gingival bleeding and excess resin around brackets are associated with higher levels of anterior gingival enlargement in subjects under orthodontic treatment.

## Abstract (pt)

**Objetivo:**

o objetivo desse estudo foi verificar a associação entre volume gengival (AG) com condições periodontais e características sócio-demográficas em sujeitos com aparelho ortodônticos fixo.

**Métodos:**

uma amostra, de 330 participantes com aparelho ortodôntico fixo, por pelo menos seis meses, foi examinada, por um único examinador calibrado, para os índices de placa e gengivais, profundidade de sondagem, nível de inserção clínico e aumento de volume gengival. O *status* socioeconômico, tempo com aparelho ortodôntico fixo e uso de fio dental foram verificados por entrevista oral. A verificação das associações foi realizada por meio de modelos de regressão de Poisson sem ajuste e ajustados.

**Resultados:**

a presença de sangramento gengival (RR 1.01; 95% IC 1.00-1.01) e o excesso de resina em torno dos braquetes (RR 1.02; 95% IC 1.02-1.03) foram associadas a um aumento do AG. Não foram encontradas associações entre características sócio-demográficas e AG.

**Conclusão:**

sangramento gengival proximal na região anterior e excesso de resina no entorno dos braquetes estão associados a níveis mais altos de aumento de volume gengival na região anterior em sujeitos com aparelho ortodôntico fixo.

## INTRODUCTION

The balance of health-disease processes in Periodontics depends on adequate supra and subgingival plaque control achieved by patient and professional's combined efforts. Accumulation of supragingival plaque results in inflammatory alterations of gingival tissues. However, interindividual differences might explain the different patterns of response and time needed for evident clinical responses. It is possible that these variations may be associated with different plaque growth patterns, local and systemic individual resistance or even a specific microbial challenge.^1,2^

Clinical studies suggest that orthodontic treatment may be associated with a decrease in periodontal health.^3,4,5^ One of the adverse periodontal alterations is a hypertrophic form of gingivitis.^5,6,7^ The exact mechanism for the development of gingival enlargement (GE) is not yet completely understood, but it probably involves increased production by fibroblasts of amorphous ground substance with a high level of glycosaminoglycans. Increases in mRNA expression of type I collagen and up-regulation of keratinocyte growth factor receptor could play an important role in excessive proliferation of epithelial cells and development of GE.^8^ In some studies, poor oral hygiene increased GE.^9,10^ Other clinical studies concluded that overall gingival changes during orthodontic treatment are transient with no permanent damage to the periodontal supporting tissues.^11,12^

The presence of orthodontic brackets also increases the skills and effort required to maintain good levels of oral hygiene, especially on the proximal surfaces.^11^ Microbiological studies demonstrate that when fixed orthodontic appliances are placed, the potential for quantitative^13,14^and qualitative^15,16^ changes in the microbial composition of these areas enhances. Thus, periodontal reaction might be elicited by a change in the microbiological environment.

To the best of our knowledge, there are no studies assessing GE and associated factors in individuals undergoing orthodontic therapy. Thus, this study aimed at assessing the prevalence of GE and associated factors in a group of orthodontic patients.

## MATERIAL AND METHODS

This cross-sectional study examined subjects who were undergoing orthodontic treatment in an orthodontic graduate program in Santa Maria, Brazil. Ethical approval was obtained from the Franciscan University Center Institutional Review Board prior to the start of the study (protocol registration number 1246 in the National Ethics Committee). Subjects who agreed to participate signed an informed consent form. Patients diagnosed with oral pathological conditions were advised to seek consultation and treatment.

### Eligibility criteria

To be eligible for the study, individuals should have been undergoing fixed orthodontic treatment for at least 6 months. Exclusion criteria comprised presence of diseases and conditions that could pose health risks to the participant or that could interfere in clinical examination, for instance, users of nifedipine, cyclosporine or phenytoin, contraceptive and decompensated diabetics. Individuals who had undergone antibiotic therapy within three months prior to examination were also excluded. Female subjects were excluded if they were pregnant or breastfeeding. Additionally, individuals who required a prophylactic regimen of antibiotics for clinical examination were excluded.

### Study sample

The orthodontic dental clinic of Ingá College (UNINGÁ) was treating, during data collection period, an estimated population of seven hundred patients, six hundred of which were regularly under treatment and, therefore, were assessed for eligibility criteria. This process resulted in four hundred eligible individuals, 330 of which were assessed, resulting in a non-response rate of less than 20%. Clinical examinations were performed between September/2009 and July/2010.

### Examinations

A single, calibrated examiner performed all clinical examinations with the aid of a dental assistant who took the records. All permanent and fully erupted teeth, except for third molars, were examined by means of a manual periodontal probe (Neumar^®^, São Paulo,SP, Brazil). Six sites (mesio-buccal, mid-buccal, disto-buccal, disto-lingual, mid-lingual, and mesio-lingual) were assessed for each tooth.

Teeth in each quadrant were washed with air and water spay as well as dried with air spray. Afterwards, Plaque Index (PII),^17^ Gingival Index (GI),^18^ probing depth (PPD), attachment loss (CAL) and bleeding on probing were assessed. Thereafter, gingival enlargement^19^ was assessed. The degree of gingival thickening in both labial and lingual aspects was scored as follows: 0 = normal; 1 = thickening up to 2 mm; 2 = thickening greater than 2 mm. The extent of gingival tissues encroachment onto the adjacent crowns was also graded, using 0, 1, 2 and 3 on the labial and lingual surfaces. The sum of both scores (thickening and gingival encroachment) resulted in an enlargement score for each gingival unit. The maximum score obtainable using this method is 5. Additionally, excess resin was dichotomously assessed by inspection with a probe around the bracket on the buccal surfaces of each bonded bracket. For this purpose, the buccal surface was divided into distal, mesial and cervical. Excess resin, located at less than 1 mm from the gingival margin was present.

After clinical examination, socioeconomic and demographic data were collected using a structured written questionnaire. Race was scored as white or non-white. Socioeconomic status was scored by the individual's level of education (≤ 11 years / > 11 years) which, in Brazil, corresponds to those who have completed high school or those educated beyond high school level. Household income information was measured in terms of the Brazilian minimum wage, which corresponded to approximately US$290 (US dollars) during the period of data collection. Income information was measured dichotomously (≤ 5 national minimum wages / > 5 national minimum wages). PII and GI were dichotomized as visible plaque (present / absent) and gingival bleeding^20^(present / absent), respectively. The percentages of sites with visible plaque, gingival bleeding and bleeding on probing were calculated individually. The questionnaire also reported the declared frequency dental floss use. Regular interdental hygiene was defined as the use of dental floss at least once a day. Non-users of dental-floss were defined as subjects who did not use interdental oral hygiene devices every day or who did not perform interdental hygiene.

### Measurement reproducibility

The examiner was trained and calibrated to perform the clinical measurements before the study started. Assessment of measurement reproducibility was conducted with 15 subjects who were divided into three groups of 5 subjects each. In each one of the groups, replicate measurements were made by the examiner on two occasions, with a two-day interval. At the site level, reproducibility was assessed by means of the weighted Kappa (± 1 mm) and resulted in values of 0.73 for probing pocket depth and of 0.68 for clinical attachment loss. Gingival enlargement reproducibility was assessed by means of Intraclass Correlation coefficient at the site level in 15 subjects, resulting in an ICC of 0.86.

### Data analysis

Most participants presented low mean values for PPD and CAL. Thus, these data were not used in the present analysis. For this exploratory analysis, data pattern distribution was analyzed and non-parametric (Mann-Whitney and Kruskal-Wallis) tests were used. After descriptive analysis, the frequencies of gingival enlargement scores (full mouth mean) were compared for differences between demographic characteristics, socioeconomic indicators and clinical status.

Unadjusted Poisson regression analysis with robust variance was performed to correlate the overall mean of the anterior gingival enlargement (AGE) score with each demographic, socioeconomic and clinical indicator. AGE was considered for anterior teeth located in esthetic regions, only. This region was chosen because a recent publication^21^ emphasized the impact of AGE in oral health related quality of life (OHRQoL) in subjects undergoing fixed orthodontic treatment. In this analysis, the outcome was considered as continuous. Additionally, rate ratios (RR), which correspond to the quotient between average scores of each comparison group, and 95% confidence intervals (95% CI) were calculated. A multivariate model was then run with the covariates. These covariates were selected using a backward stepwise procedure. Only variables with p ≤ 0.20 or those that presented a conceptual association with the primary outcome were included in the model. In order to be retained in the final multivariate model, the variables should present p ≤ 0.05. The statistical software STATA 9.0 (Stata Corp, College Station, USA) was used for all analyses.

## RESULTS

All subjects completed both the questionnaire and the clinical examination. The demographic characteristics, socioeconomic indicators and clinical status of the subjects are shown in Table 1. In the present investigation, the study sample comprised 330 individuals aged between 14 and 30 years old, 171 (51.8%) female and 159 (48.2%) male, 263 (79.7%) white and 67 (20.3%) non-white. As for periodontal diagnosis, based on patient's age and periodontal data explored in another study^22^ (2.06 mm ± 0.18 and 1.6 ± 0.11 for mean probing depth and clinical attachment levels in proximal sites, respectively) most patients had periodontal diagnosis of gingivitis, only.

**Table 1:** Research subjects' clinical and socio-demographic characteristics.

Variables	n (%)
**Demographic characteristics**	
**Sex**	
Male	159 (48.1%)
Female	171 (51.8%)
**Ethnicity**	
White	263 (79.7%)
Non-White	67 (20.3%)
**Age (years)**	
14 - 19	162 (49.0%)
20 - 24	121 (36.6%)
25 - 30	47 (14.2%)
***Socioeconomic status***	
**Household Income**	
≤ 5	270 (81.8%)
> 5	60 (18.1%)
**Educational level**	
> 11 years	159 (48.1%)
≤ 11 years	171 (51.8%)
***Clinical status***	
**TUFOT (Months)**	
6 - 12	185 (56.06%)
> 12	145 (43.93%)
**Dental floss**	
Users	81 (24.5%)
Non-users	249 (75.5%)

TUFOT: Time under fixed orthodontic treatment.

The mean value for anterior gingival enlargement, percentage of whole mouth visible plaque and percentage of whole mouth gingival bleeding were 0.69, 47.38% and 58.72%, respectively. Table 2 shows the unadjusted analysis between demographic, socioeconomic and clinical variables related to different scores of gingival enlargement. Statistically significant differences were observed in each frequency of gingival enlargement for almost all covariates. Level of education was not associated with GE. When considering score 2 of GE, no association was detected with sex and for score 3 of GE, age was not associated.

**Table 2:** Univariate analysis between socioeconomic factors and oral clinical conditions related to frequencies of different scores of gingival enlargement index.

Variables	N (%)	GE0 (%)	GE1 (%)	GE2 (%)	GE3 (%)
		Mean ± SD	Mean ± SD	Mean ± SD	Mean ± SD
**Demographic characteristics**					
**Sex**		p* = 0.010	p* = 0.010	p* = 0.120	p* < 0.001
Male	159 (48.1%)	58.10 ± 9.24	27.56 ± 4.55	12.52 ± 8.95	1.21 ± 3.43
Female	171 (51.8%)	58.15 ± 9.66	29.05 ± 5.53	10.48 ± 8.20	1.70 ± 2.67
**Ethnics (%)**		p* < 0.001	p* = 0.016	p* = 0.001	p* = 0.000
White	263 (79.7%)	59.17 ± 10.0	28.05 ± 5.04	11.17 ± 9.35	1.10 ± 3.19
Non-white	67 (20.3%)	54.04 ± 5.01	29.16 ± 5.23	13.0 ± 4.72	2.80 ± 2.21
**Age (yrs)**		p** = 0.037	p** < 0.001	p** < 0.001	p** = 0.083
14 - 19	162 (49.0%)	56.98 ± 10.15	27.59 ± 4.86	12.89 ± 9.19	1.88 ± 3.74
20 - 24	121 (36.6%)	59.88 ± 9.36	29.52 ± 5.30	8.91 ± 7.66	1.05 ± 2.51
24 - 30	47 (14.2%)	57.57 ± 5.79	27.46 ± 4.82	13.63 ± 7.51	0.95 ± 1.25
**Socioeconomic status**					
**Household income (Wages)**		p* < 0.001	p* < 0.001	p* < 0.001	p* < 0.001
≤ 5	270 (81.8%)	56.62 ± 8.72	27.55 ± 4.39	13.35 ± 8.25	1.77 ± 3.33
> 5	60 (18.1%)	64.88 ± 9.61	31.56 ± 6.60	3.36 ± 4.81	0.0 ± 0.0
**Subject's education**		p* = 0.055	p* < 0.001	p* = 0.011	p* < 0.001
≤ 11 years	159 (48.1%)	57.28 ± 9.91	27.51 ± 4.73	13.03 ± 9.11	1.46 ± 3.63
> 11 years	171 (51.8%)	59.41 ± 8.54	29.45 ± 5.41	9.28 ± 7.35	1.42 ± 2.03
**Clinical status**					
**Dental floss users**		p* = 0.018	p* < 0.001	p* < 0.001	p* = 0.031
Yes	81 (24.5%)	61.46 ± 8.28	31.25 ± 5.64	6.44 ± 6.66	0.45 ± 0.90
No	249 (75.5%)	57.04 ± 9.54	27.31 ± 4.51	13.2 ± 8.57	1.77 ± 3.46
**TUFOT (Months)**		p* <0.001	p* <0.001	p* <0.001	p* =0.016
6 a 12	185 (56.06%)	60.56 ± 6.40	29.26 ± 3.77	8.78 ± 3.66	0.81 ± 1.69
> 12	145 (43.93%)	55.02 ± 11.56	27.02 ± 6.18	15.02 ± 11.46	2.26 ± 4.13

Caption :GE0=Gingival enlargement score 0; GE1=Gingival enlargement score 1; GE2=Gingival enlargement score 2; GE3=Gingival enlargement score 3; TUFOT: Time under fixed orthodontic treatment;

p*= Mann Whitney test;

p**= Kruskall Wallis test

The unadjusted Poisson regression assessment of associations revealed use of dental floss (RR 1.25; 95% CI 1.08-1.43), percentage of proximal anterior gingival bleeding (RR 1.01; 95% CI 1.0-1.01) and percentage of sites with excess resin around brackets (RR 1.02; 95% CI 1.02-1.03) as the main covariates associated with higher levels of anterior gingival enlargement. In the multivariate regression model, the percentage of proximal anterior gingival bleeding remained associated, in which higher frequencies of anterior gingival bleeding were associated with a 1.01 fold increase in average AGE scores (RR 1.01; 95% CI 1.0-1.01). Additionally, the percentage of sites with excess resin around brackets also remained associated, with higher frequencies of sites with excess resin around brackets being associated with a 1.02 fold increase in average AGE scores (RR 1.02; 95% CI 1.02-1.03).

## DISCUSSION

The present study aimed at investigating potential associations among gingival enlargement, socio-demographic and clinical characteristics. When GE was assessed by frequency of scores, statistically significant differences between nearly all independent variables and frequencies of GE were observed. While the clinical relevance of these differences could be dubious for some of these variables, others such as household income, use of dental floss and time with orthodontic appliances presented substantial clinical differences for the prevalence of gingival enlargement score two. After regression analysis had been performed, proximal anterior gingival bleeding and excessive resin around brackets were revealed as independent variables associated with the level of anterior gingival enlargement.

Since hyperplasic gingival response is a common response to plaque accumulation in subjects undergoing fixed orthodontic therapy. this study used two indexes to assess gingival inflammatory status: color alteration and/or swelling and bleeding after marginal probing. Gingival bleeding IS shown by clinical and histological studies to be an earlier and more sensitive sign of gingival inflammation in comparison to cardinal signs such as redness and swelling, which may be rather subjective and not very reliable.^23,24^ Furthermore, a single calibrated examiner, unaware of dental floss use habits, assessed all parameters. These methods probably increased the quality of data collection as well as results reliability.

The use of dental floss was dichotomized into subjects who use it every day and individuals who do not have this habit. This cutoff was made based on evidence that demonstrates reduction in inflammatory parameters associated with gingivitis when flossing is performed every day.^25^ The use of dental floss was not an independent predictor for AGE. There is no evidence in population basis evaluating the association between dental floss and GE. However, short-term clinical studies with subjects without orthodontic appliances have demonstrated a significant improvement in the interproximal gingival condition with the correct use of dental floss.^25,26,27^ It is important to highlight that some studies conducted with highly motivated individuals who efficiently brush their teeth revealed that the incorporation of flossing in oral hygiene routines does not significantly contribute to improve interproximal gingival conditions.^28^ Thus, the lack of association between dental floss and AGE may be due to the possibility of toothbrushing alone resulting in low levels of plaque accumulation in anterior segments. These results should be interpreted with care. Although no statistically significant differences were observed in AGE in users and non-users of dental floss, this habit is also recommended for other purposes, such as preventing attachment loss, halitosis, carious lesions, etc.

The positive correlation found between sites with excess resin around brackets and levels of AGE confirmed the hypothesis that oversight in the bonding of brackets might influence gingival enlargement. The standard of finishing/polishing techniques and surface roughness proved to be important factors for bacterial adhesion with different types of dental materials.^29,30^ Thus, our results could hypothesize that excess resin causes greater adhesion of bacterial plaque and subsequent gingivitis formation. Moreover, poor oral hygiene proved to be an important causal factor of gingival enlargement.^9^

In this study, variables related to socio-demographic characteristics, such as sex, ethnicity, household income and subjects' level of education, were not associated with anterior gingival enlargement. However, it has been established that individuals from different socioeconomic backgrounds are exposed to different risk factors that affect oral health. Individuals with lower socioeconomic status are subjected to material deprivation which could influence their engaging in riskier behaviors, thereby resulting in worse oral health conditions.^31^ Furthermore, low educational level may lead to reduced income, unemployment and poor occupational status, all of which could influence oral health.^31^ Our contradictory results could be explained by one of the following hypotheses: First, in subjects with orthodontic appliances, socio-demographic characteristics may not influence gingival enlargement levels, differently to subjects without orthodontic appliances; second, the lack of extreme differences related to socioeconomic status in our sample may have influenced the results, i.e., individuals participating in this study were recruited at a private institution, presenting at least six years of education without a great disparity between educational levels.

Studies assessing the association between levels of anterior gingival enlargement and socioeconomic as well as clinical conditions in orthodontic subjects (using multiple regression analyses controlled for other socio-demographic and clinical variables which may act as confounders) are inexistent. From this perspective, the present study provides new information. In addition, Poisson regression with robust variance was used in order to provide PR estimates which are easier to interpret than odd ratios. In a situation of anterior gingival enlargement, with prevalence higher than 50%, odd ratios would strongly overestimate PRs.^32^ It is important to highlight that this study had a cross-sectional design, which hypothesizes relations between the outcome and predictor variables without establishing causal relationships. This is a limitation of this study. However, conclusions from cross-sectional studies are important to identify indicators that may be included in longitudinal or, even, experimental studies. The present study comprised 330 orthodontic patients attending a private orthodontic specialist training program. This sample limited the extent to which these findings can be generalized to a larger population. Nevertheless, analyses were performed with sufficient power and the analytical results strengthen the hypotheses of this study.

Evidence shows that gingival enlargement is associated with esthetic impairment and, in more severe cases, with phonetic alterations and masticatory problems.^7^ A recent publication^21^ conducted with an orthodontic sample emphasized the impact of anterior gingival enlargement in oral health related quality of life (OHRQoL). Thus, prevention and/or treatment of gingival enlargement may contribute to improve the OHRQoL of orthodontic patients. According to our results, proximal anterior bleeding and excess resin around brackets were associated with higher levels of anterior gingival enlargement. However, further studies are required to understand whethwe it is possible that prevention and treatment of gingivitis and careful bonding of brackets may result in decrease in the prevalence or even the severity of GE in orthodontic subjects.

## CONCLUSIONS

Anterior gingival enlargement is associated with gingival inflammation and excess resin around brackets.

## Figures and Tables

**Table 3:** Unadjusted (RR) and Adjusted assessment (RR*) for association between socioeconomic factors and oral clinical conditions related to anterior gingival enlargement average. Robust poisson regression analysis.

Variables	n (%)	AGE	AGE	AGE
		Mean ± SD	RR (95%IC)	RR* (95% IC)
**Demographic characteristics**				
**Sex**			p = 0,142	**
Male	159 (48.1%)	0.72 ± 0.33	1.08 (0.97-1.20)	
Female	171 (51.8%)	0.66 ± 0.34	1	
**Ethnics (%)**			0.188	**
White	263 (79.7%)	0.7 ± 0.34	1	
Non-white	67 (20.3%)	0.64 ± 0.32	0.91 (0.79-1.04)	
**Age (yrs)**			p = 0.123	**
14-19	162 (49.0%)	0.73 ± 0.35	1.11 (0.96-1.29)	
20-24	121 (36.6%)	0.66 ± 0.34	1.00 (0.85-1.17)	
24-30	47 (14.2%)	0.65 ± 0.29	1	
**Socioeconomic status**				
**Household income (Wages)**			p = 0.213	**
≤ 5	270 (81.8%)	0.70 ± 0.33	1.10 (0.94-1.28)	
> 5	60 (18.1%)	0.64 ± 0.35	1	
**Subject's education**			p = 0.14	**
< 11 years	159 (48.1%)	0.73 ± 0.35	1.08 (0.97 – 1.20)	
≥ 11 years	171 (51.8%)	0.63 ± 0.31	1	
**Clinical status**				
**Dental floss users (%)**			p = 0.000	p = 0.239
Yes	81 (24.5%)	0.58 ± 0.34	1	1
No	249 (75.5%)	0.73 ± 0.33	1.25 (1.08-1.43)	1.07 (0.95-1.22)
**TUFOT (Months)**			p = 0.18	**
6 a 12	185 (56.06%)	0.64 ± 0.32	1	
> 12	145 (43.93%)	0.75 ± 0.35	0.91 (0.79-1.04)	
PAGB (%)		-	p = 0.000	p = 0.000
			1.01(1.0 – 1.01)	1.01 (1.00-1.01)
**Excessive resin (% of sites)**		-	p = 0.000	p = 0.000
			1.02 (1.02-1.03)	1.02 (1.02-1.03)

RR: Ratios rate; AGE: Anterior gingival Enlargement; TUFOT: Time under fixed orthodontic treatment; PAGB: Proximal anterior gingival bleeding

* Adjusted by sex, ethnics, age, Household income, subjects´s education, TUFOT, dental floss, GAE and excessive resin

** Variables not included in the final multiple model after adjustment.
